# Assessment of Factors Responsible for Stone-Free Status After Retrograde Intrarenal Surgery

**DOI:** 10.7759/cureus.63627

**Published:** 2024-07-01

**Authors:** Kishan Raj K, Prashant Adiga K, Reshmina Chandni Clara D'souza, Nandakishore B, Manjunath Shetty

**Affiliations:** 1 Urology, Father Muller Medical College and Hospital, Mangalore, IND; 2 General Surgery, Father Muller Medical College and Hospital, Mangalore, IND; 3 Urology, Malabar Medical College Hospital and Research Centre, Kozhikode, IND

**Keywords:** stone-free rate, renal infundibulopelvic angle, renal infundibular length, resorlu-unsal stone score, rirs score

## Abstract

Aim

The study aimed to evaluate the predictive factors that determined stone-free rate (SFR) after retrograde intrarenal surgery (RIRS).

Materials and methods

This prospective study was conducted on 183 patients undergoing RIRS for renal stones. Patients were categorized into two groups, depending on stone-free status one month following the procedure. SFR was defined as the complete absence of stones or stones <4 mm. The parameters studied included patient demographics, presence of hydronephrosis, presence of preoperative double J-stent, abnormal renal anatomy, and stone characteristics (stone burden, stone number, stone density, stone location, lower pole infundibulopelvic angle, and lower pole renal infundibular length (RIL)). Univariate and multivariate analyses were performed to identify risk factors for residual stones. We assessed the predictive ability of the RIRS score and Resorlu-Unsal stone score (RUSS) for evaluating SFR utilizing receiver operating characteristic (ROC) analysis.

Results

183 patients were included in the study with a median age of 51 years. 131 (71.6%) patients were declared stone-free after the procedure. The mean stone size and density were 16.9 SD±7.5 mm and 1038 SD±342 Hounsfield units (HU) respectively. Stone-free patients had lower stone size (14.3 mm vs. 23.6 mm, p<0.01) and stone density (970 HU vs. 1211 HU, p<0.01) compared to non-stone-free patients. Patients with residual stones had steeper lower pole renal infundibulopelvic angle (RIPA) (31.3° vs. 40.7°, p<0.01) and longer RIL (26.6 mm vs. 21.1 mm, p<0.01). Stone multiplicity (p<0.01), lower pole stone location (p<0.01), and renal malformations (p<0.01) were significant influencing factors for residual renal stones after RIRS. Multivariate analysis revealed stone size, stone density (HU), and stone location as independent predictors for SFR after RIRS. Among the scoring systems, the RIRS score had the highest diagnostic accuracy for SFR (area under the curve (AUC): -0.882, 95% CI-0.828-0.936).

Conclusion

Stone size, stone density (HU), and stone number are important predictors of SFR after RIRS. Lower pole stone location and abnormal renal anatomy play a substantial role in determining SFR after RIRS. In lower pole stones, a long RIL and acute RIPA negatively influence SFR. Additionally, the RIRS score was found to be a better predictor for SFR than the RUSS score.

## Introduction

Kidney stone disease is a common urological problem affecting nearly 12% of the world's population at some point in their lifetime [1]. Globally, the prevalence rate of stone disease is increasing due to changes in dietary habits and lifestyle modifications. The disease is most common in the age group between 20 and 49 years and is more common in males than in females [2].

Multiple competing treatment modalities exist for symptomatic renal stones. Retrograde intrarenal surgery (RIRS) is gradually gaining popularity due to its low risk of complications compared to percutaneous nephrolithotomy (PCNL). Although American Urology Association (AUA) guidelines recommend PCNL as the standard option for stones >2 cm, it cautions that the advantages be closely weighed against increased invasiveness and complications of PCNL [3]. Compared to RIRS, PCNL resulted in a more significant decrease in postoperative hemoglobin and a higher risk of complications [4]. Innovations in flexible ureteroscopy have popularized RIRS in patients with abnormal renal anatomy, musculoskeletal deformities, and bleeding diathesis [5].

The success of RIRS is determined by the stone-free rate (SFR), auxiliary procedures needed, and complications. Multiple diverse factors govern SFRs in RIRS, such as stone burden, stone density, stone location, number of stones, and calyceal anatomy [6]. Reported SFR rates for RIRS vary from 54% to 96% for renal stones smaller than 2 cm [7]. This wide variation is probably due to multiple competing variables that influence SFR.

Multiple nomograms and scoring systems have been developed to predict successful stone clearance after RIRS. These scoring systems help in preoperative patient counseling and guide management decisions. Resorlu-Unsal stone score (RUSS) score is a simple and quick scoring system to predict SFRs after RIRS [8]. RIRS score is a new and innovative scoring system based on stone density, renal infundibular length (RIL), renal infundibulopelvic angle (RIPA), and stone burden [9].

We aim to analyze various factors that contribute to SFR in RIRS and compare RIRS and RUSS scoring systems for determining SFR.

## Materials and methods

Study design and population

The current study was undertaken at Father Muller Medical College, Mangalore, India, between June 2023 and May 2024, after approval by the Father Muller Institutional Ethics Committee (FMIEC/CCM/375/2023). It was conducted in accordance with the Declaration of Helsinki after obtaining written informed consent from all the participants.

The study included all patients over 18 years with renal stones. Patients with stone sizes between 10 mm and 35 mm were incorporated into the study. Patients with a history of renal stone surgeries in the past and with preoperative double J (DJ) stent were enrolled. The study included patients with musculoskeletal malformations or anatomic abnormalities of the kidney. The study excluded pregnant patients and patients with active urinary tract infection (UTI) and severe immunodeficiency.

Information regarding patient demographics (age, sex, history of renal stone surgery, and preoperative DJ stent) was collected. CT urogram was performed to look for stone location, number, and stone density. The infundibular length was calculated from the distalmost point of stone bearing calyx to the mid-point of the renal pelvis. The infundibulopelvic angle of the lower pole stone was defined as the inner angle of the intersection of the renal pelvic axis and the axis of the lower pole calyx. The stone burden was calculated by measuring the maximum cumulative diameter of all renal and ureteric stones. RIPA and RIL were measured only for lower pole stones.

Every case was classified and graded based on RUSS score after considering four factors. Each factor had been given a score of one point each: stone size of >20 mm; lower pole position with RIPA <45°; stone number in various calyces more than one; and aberrant renal anatomy [8]. The total score ranged from one to four.

The RIRS scoring system was developed by Xiao et al. in 2017 [9]. The score is calculated based on the following criteria: renal stone density ≤1000 Hounsfield units (HU) (one point) or >1000 HU (two points), inferior pole stone with RIPA (scored from one to three points as determined by a non-inferior pole stone or inferior pole stone with RIPA >30° or ≤30°), RIL ≤25 mm (one point) or >25 mm (two points), stone burden (one to three points according to cumulative stone size of ≤10 mm, >10 mm and ≤20 mm, and >20 mm, respectively).

SFR was defined as the absence of residual fragments larger than 4 mm one month after the procedure. Stone-free status was documented with a combination of abdominal ultrasound (USD) and abdominal X-ray in radio-opaque stones and a non-contrast CT scan in radiolucent stones. Patients with residual fragments requiring further RIRS were routinely scheduled for elective second treatment. 

Operative technique

All procedures were performed in a standard lithotomy position under spinal anesthesia by two surgeons with experience of >100 cases each. Patients with positive urine cultures and those with preoperative UTI were adequately treated with appropriate antibiotics before the procedure. RIRS was performed only after obtaining a sterile urine culture. Before the start of the procedure, patients received prophylactic preoperative antibiotic prophylaxis in accordance with our hospital guidelines.

After the initial cystoscopy, a 0.035 standard nitinol-coated polytetrafluoroethylene (PTFE) body guide wire was inserted as a safety wire into the renal pelvis. A preliminary ureteroscopy with a 7 Fr semirigid ureteroscope (Karl Storz, Germany) was performed to evaluate for the presence of stones and confirm compliance of the ureter. 9.5 Fr ureteric access sheath (Cook Medical, Bloomington, IN, USA) was inserted under fluoroscopic control with its tip below the pelvic-ureteric junction. In case of difficult insertion due to narrow or non-compliant ureter, DJS was inserted, and RIRS was postponed for two weeks. Flexible ureteroscopy was conducted using a 7.5 Fr flexible digital ureterorenoscope (Biorad Medisys, India). In lower pole stones, stones were engaged with a 2.2 Fr zero tip nitinol basket (Cook Medical, Bloomington, IN, USA) and brought to the renal pelvis before fragmentation. Lithotripsy was done with 200 micron fiber utilizing a 30-watt Holmium yttrium-aluminum-garnet laser (Quanta, Italy). A meticulous inspection of the renal collecting system was done at the end to confirm stone clearance. DJS was routinely inserted after every procedure.

Statistical methods

Patient demographic data and stone variables were collected prospectively. Continuous variables were expressed in terms of mean ± SD or median (Q1-Q3; interquartile range). Categorical data were presented by n (%). The Chi-square test was used to assess the categorical variables. The normality of continuous data was checked using the Kolmogorov-Smirnov test. Student t-test or Mann Whitney U test were used to analyze continuous variables between those with and without residual stones. Multivariate binary logistic regressions were used to identify independent risk factors for SFR after RIRS. ORs with 95% CI was calculated. The predictive ability of RUSS and RIRS scoring systems to accurately predict SFR was evaluated by the area under the receiver operating characteristic (AUROC) curve. Statistical analysis was performed using SPSS version 23 (IBM Corp., Armonk, NY). All p-values were two-sided, and a p-value of <0.05 was regarded as statistically significant.

## Results

Out of 183 patients who underwent RIRS, stone-free status was noted in 131 (71.6%) patients. Patient demographic details and stone characteristics are depicted in Table 1. The majority of patients were male (61.2%). The median age of the patients in the cohort was 51 years, and there was no statistical difference in the age group of patients who did and did not become stone-free after RIRS (p: 0.22). The presence of hydronephrosis, history of renal stone surgery, and preoperative DJ stent did not significantly influence SFR after RIRS. Patients with multiple renal stones and with lower pole stone locations were found to have a higher likelihood of residual stones in the postoperative period. Stone-free patients were noted to have smaller stone size (14.3 mm vs. 23.6 mm, p<0.01) and lower stone density (970 HU vs. 1211 HU, p<0.01). In contrast, lower pole stones with acute RIPA (31.1° vs. 40.7°, p<0.01) and longer RIL (26.6 mm vs. 21.1 mm, p<0.01) had lower SFR in the postoperative period.

**Table 1:** Clinical and demographic characteristics of patients grouped according to stone-free status RIPA: renal infundibulopelvic angle; RIL: renal infundibular length; RIRS: retrograde intrarenal surgery; RUSS: Resorlu-Unsal stone; IQR: interquartile range

Variable	Total (%)	Stone free (%)	Residual stone (%)	p-value
Number of patients	183	131 (71.6%)	52 (28.4%)	
Age in years (median, IQR)	51 (39-59)	50 (38-59)	53 (45-58.5)	0.22
Female sex	71 (38.8%)	46 (64.8%)	25 (35.2%)	0.11
Presence of hydronephrosis	62 (33.8%)	42 (67.7%)	20 (32.3%)	0.41
Prior renal stone surgery	38 (20.8%)	23 (60.5%)	15 (39.5%)	0.09
Preoperative DJ stent	75 (40.9%)	59 (78.7%)	16(21.3%)	0.08
Multiple stones	63 (34.4%)	30 (47.6%)	33 (52.3%)	<0.01
Lower pole location	86 (47%)	48 (55.8%)	38 (44.2%)	<0.01
Abnormal renal anatomy	10 (5.4%)	3 (30%)	7 (70%)	<0.01
Stone size (mm) (mean±SD)	16.9±7.5	14.3	23.6	<0.01
Stone HU (mean±SD)	1038±342	970	1211	<0.01
RIPA (°) (mean±SD)	36.4±12.3	40.7	31.3	<0.01
RIL (mm) (mean±SD)	23.6±6	21.1	26.6	<0.01
RIRS score (median, minimum-maximum)	6 (4-10)	6 (4-9)	8 (5-10)	<0.01
RUSS score (median, minimum-maximum)	1 (0-4)	0 (0-3)	2 (0-4)	<0.01

Multivariate logistic regression analysis was done to identify independent predictors for stone-free outcomes. Predictors with statistically significant associations in univariate analysis were selected for multivariate analysis. Additionally, since RIPA and RIL were measured for only those with lower pole stones, they were not included in the logistic regression analysis. Multivariate logistic regression analysis (Table 2) revealed stone size (OR: 0.828, p<0.01), stone density (0.997, p<0.01), and lower pole stone location (OR: 0.363, p: 0.05) to be strong predictors of residual stones following RIRS.

**Table 2:** Multivariate logistic regression analysis to predict SFR RIRS: retrograde intrarenal surgery; RUSS: Resorlu-Unsal stone; HU: Hounsfield unit; SFR: stone-free rate

Variable	OR (95% CI)	p-value
Stone size (mm)	0.828 (0.758-0.905)	<0.01
Stone HU	0.997 (0.995-0.998)	<0.01
Lower pole stone location	0.363 (0.135-0.977)	0.05
Multiple renal stones	1.303 (0.136-12.481)	0.82
Abnormal renal anatomy	0.395 (0.057-2.721)	0.35
RIRS score ≥6	0.637 (0.070-5.815)	0.70
RUSS score ≥2	0.348 (0.034-3.561)	0.38

**Figure 1:**
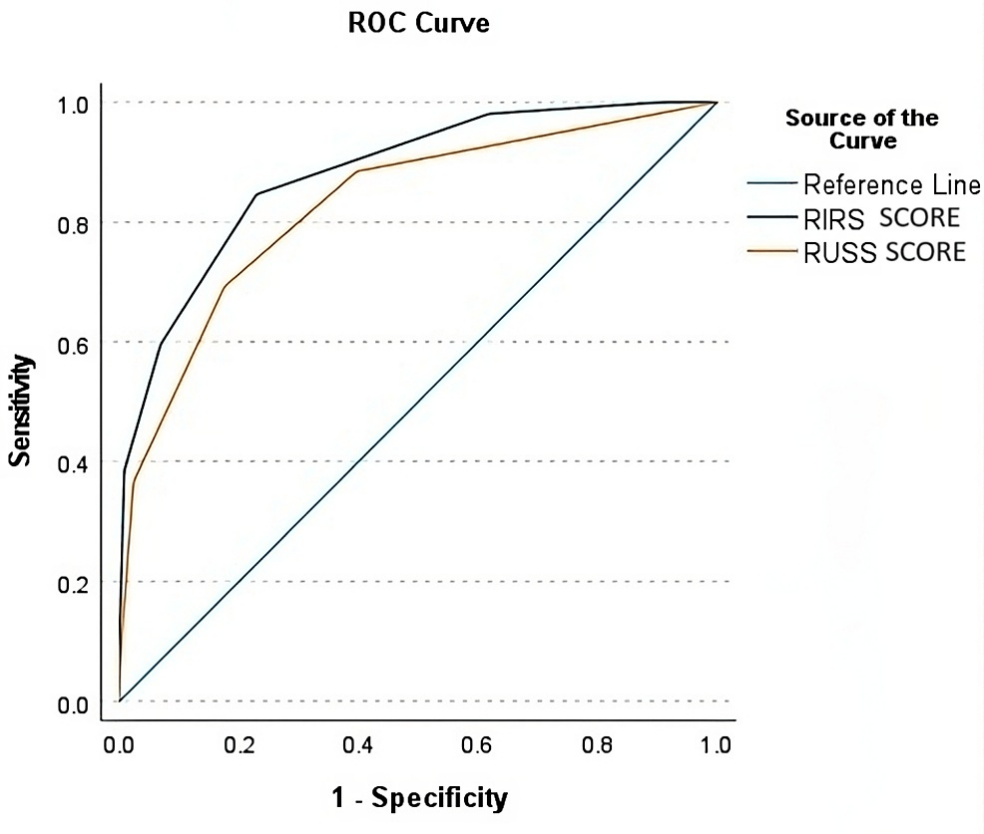
ROC curve comparing RIRS score and RUSS score RIRS: retrograde intrarenal surgery; RUSS: Resorlu-Unsal stone; ROC: receiver operating characteristic

Receiver operating characteristic (ROC) curve analysis was carried out to determine the diagnostic accuracy of RIRS and RUSS scoring systems to predict SFR. The ROC curve is illustrated in Figure 1. The results revealed a significant area under the curve (AUC) for RIRS score (AUC: 0.882 95% CI: 0.828-0.936, p<0.00) and RUSS score (AUC: 0.828 95% CI: 0.759-0.897, p<0.00) (Table 3).

**Table 3:** Area under ROC curve RIRS: retrograde intrarenal surgery; RUSS: Resorlu-Unsal stone; ROC: receiver operating characteristic

Test variable	Area	Standard error	p-value	95% CI
RIRS score	0.882	0.028	<0.01	0.828-0.936
RUSS score	0.828	0.035	<0.01	0.759-0.897

## Discussion

The increasing prevalence of urolithiasis has placed a significant burden on patients and society. Extracorporeal shockwave lithotripsy (ESWL) and RIRS are the available options for treating renal stones between 1 cm and 2 cm [3]. The vast popularity of RIRS is due to technological advancements by way of endoscope miniaturization and the evolution of optics from fiber optic to digital ureteroscopy. Improved deflection in newer generation digital ureteroscopes has enabled successful endoscopic treatment of lower pole stones with steep RIPA. Stones previously tricky to clear with ESWL due to unfavorable calyceal anatomy, like long lower pole calyx and narrow infundibulum, can be easily tackled with RIRS.

Few studies have compared RIRS and PCNL outcomes for stone sizes >2 cm. Aboumarzouk et al. conducted a meta-analysis on SFR following RIRS for >2 cm stones and found the mean SFR to be 93.7% [10]. A recent study by Fayad et al. found higher SFR in PCNL compared to RIRS, but this difference was non-significant (p: 0.22) [11]. The same study showed that the blood transfusion rate was 8.3% in PCNL compared to 1.6% in the RIRS group (p: 0.08). Sebaey et al. conducted a randomized study comparing RIRS to PCNL in 70 patients with renal pelvic stones with sizes between 20 and 30 mm. There was no difference in SFR between the two groups (p: 0.50), but the PCNL group had lower mean postoperative hemoglobin levels (p: 0.07) [12]. These studies highlight equivalent success rates in RIRS compared to PCNL in large-volume stones. Compared with PCNL in large-volume stones, RIRS patients have lower blood loss, experience lesser pain, and have shorter hospital stays.

RIRS is preferred over PCNL in patients with morbid obesity, bleeding disorders, and anatomically abnormal kidneys [13]. Obtaining percutaneous access becomes difficult in patients with horseshoe kidneys, and there is a theoretically increased risk of bleeding due to aberrant vasculature. In their study that evaluated RIRS vs. PCNL in horseshoe kidneys, Ding et al. found comparable success rates for RIRS with lower complication rates [14]. RIRS is a reliable and effective modality for lithotripsy in patients with anticoagulants. Alazaby et al. reported successful outcomes of RIRS without cessation of anticoagulants [15]. PCNL in morbidly obese patients is fraught with challenges in patient positioning and fluoroscopic imaging for percutaneous access. They present with anesthetic challenges and complicate the procedure due to longer stone skin-to-stone distances and the risk of nephrostomy tube dislodgement [16]. The meta-analysis by Wang et al. has demonstrated RIRS to be a highly effective and safe modality of treatment of renal stones in morbidly obese patients [17]. RIRS has proven to be a reliable treatment modality in patients with analomous kidneys, anticoagulant medicines, and those with previous ESWL or PCNL treatment failure [15].

SFR is a crucial parameter that determines the success of RIRS. We achieved complete stone clearance in 71.6% of our patients. Schoenthaler et al. reported SFR ranging from 54% to 96% for renal stones smaller than 2 cm after one session and 86% to 92% for renal stones larger than 2 cm after one to four sessions of RIRS [7]. Another study reported stone clearance for stone size <1.5 cm as 84%, whereas stones >1.5 cm had a clearance rate of 62% [18]. Contrary to the above study, Lin et al. have found RIRS to be a safe and effective treatment option for renal stones between 2 cm and 3 cm with an acceptable SFR of 80.2% [19]. Patients with more than 3 cm stones who opted for RIRS had a lower complication rate but required staged procedures due to the lower single-session SFR (76.5%). 

The wide variation in reported SFR is due to multiple competing factors that influence SFR. There is no uniform strict definition for SFR and no modality to document it. When the USG abdomen was used to document SFR, it was found to miss small fragments and overestimate SFR [20]. CT scan is more genuine and trustworthy to document SFR due to its inherent high sensitivity. It is believed that stone fragments take time to clear spontaneously after RIRS. Studies have shown three-month SFR to be higher than one-month SFR (90.9% vs. 84.8%) [21]. We have documented SFR at one month following RIRS. Moreover, our study included relatively large volume stones. These three factors may explain why our reported SFR rates are lower.

Our study concluded that patients with a higher stone burden had significantly low SFR. Patients with residual stones had a higher stone size than those with successful stone clearance (2.36 cm vs. 1.43 cm). Multivariate analysis revealed that a 1 mL increase in the stone size was associated with a 17.2% reduction of the stone-free outcome (OR: 0.828, 95% CI: 0.758-0.905, p< .01). Goldberg et al. reported a cut of size of 15 mm, beyond which patients had lower SFR [22]. Patients with large-volume stones who undergo RIRS must be counseled regarding a staged procedure. 

According to univariate analysis, stone number and density (HU) were significant determining factors for SFR. Out of 63 patients with multiple renal stones, residual stones were found in 33 patients (52.3%, p<0.01). Patients undergoing RIRS for multiple stones were more likely to have incomplete clearance than those undergoing RIRS for solitary renal stones. Since the stone number is closely related to the cumulative stone burden, we could not find an independent association in multivariate analysis. Xiao et al. analyzed patients undergoing RIRS and found that patients with multiple stones and higher stone density had a higher probability of residual stones in follow-up [9]. Stone density is measured by Hounsefield units in a CT scan, which is closely related to stone fragility. Stones with higher HU have poor stone fragmentation efficiency and clearance rates. Our study revealed that stone-free patients have lower HU than non-stone-free patients (970 HU vs. 1211 HU, p<0.01). One HU increase in the stone density was associated with a 1% reduction of the stone-free outcome (OR: 0.998, 95% CI: 0.995-0.998, p<0.01).

RIRS in ectopic kidneys and those with high insertion of ureters represent a unique challenge. We noted that only three of 10 patients with anatomical abnormal kidneys had complete stone clearance after RIRS (30% vs. 70%, p<0.01). Insertion of the ureter into the renal pelvis is more lateral and superior in horseshoe kidneys. A higher incidence of ureteropelvic junction obstruction in horseshoe kidneys precludes satisfactory stone clearance during RIRS. The renal pelvis is comparatively flatter in these kidneys, which increases the difficulty of deflection of flexible ureteroscope during lithotripsy [23]. Ding et al. evaluated the clinical efficacy of RIRS in 16 patients with horseshoe kidneys [14]. The single-session SFR was 62.5%, and the overall SFR was 87.5%. 

RIRS in lower pole stone presents an intriguing dilemma. Inferior pole stones have been historically difficult to treat by RIRS due to limited deflection, resulting in difficult lower pole access. The anatomical challenges like acute RIPA and long narrow infundibulum decrease the spontaneous expulsion of stone fragments after lithotripsy. For this reason, AUA recommends conservative management as a practical option for asymptomatic lower pole stones [3]. However, the risk of a symptomatic stone episode is estimated to be approximately 10% per year, with a cumulative five-year event of 48.5% [24]. Xiao et al. conducted a prospective study on 573 patients undergoing RIRS. Inferior pole stones, narrow RIPA, and longer RIL were regarded as important predictors for residual stones following RIRS [9]. Ito et al. conducted a study on 238 cases of RIRS and found stone volume, stone number, and the presence of lower pole calculi to be associated with failed treatment [25]. In our study, while the overall SFR was 71.6%, SFR in lower pole stones was 55.8% (p<0.01). Multivariate analysis showed lower pole stones to negatively influence the stone-free outcome (OR: 0.363, 95% CI: 0.135-0.977, p: 0.05).

Elbahnasy et al. studied the role of pelvicalyceal anatomy impacting stone clearance after ESWL [26]. It was found that wide RIPA (≥70°), short Infundibular length (≤3 cm), and broad infundibular width (>5 mm) were positive predictors for successful stone clearance [26]. As technological advancements led to the broader applicability of RIRS in lower pole stones, the same factors that influenced stone clearance in ESWL were found to impact the success of RIRS.

It is technically challenging to achieve entry into calyces with acute IPA during flexible ureteroscopy. The dependent position of these lower pole stones hampers spontaneous passage after lithotripsy. Our analysis found a significant difference between the RIPA of stone-free and non-stone-free groups (40.7° vs. 31.3°, p<0.01). While two studies have found IPA <30° to be a negative risk factor for stone clearance, one study has suggested a cut-off of 45° [8,23,27].

RIL is another factor that is robustly associated with stone clearance. Jessen et al. studied the role of RIL and found a cut-off of 27 mm, beyond which there were high chances of residual stones [23]. Similar to this study, Grasso et al. inferred that RIL >3 cm or infundibular stricture was a significant influencing factor for stone clearance [28]. In our study, longer RIL was correlated with a higher risk of residual stones (26.6 mm vs. 21.1 mm, p<0.01).

Recently, scoring systems have come into vogue to predict successful stone clearance. These scoring systems combine multiple criteria with different weightages and predict the probability of successful stone clearance. They can guide preoperative counseling and help select an appropriate operative modality. RUSS is one of the earliest developed scoring systems to predict SFR after ureteroscopic lithotripsy [8]. It is based on stone size, stone number, lower pole stone location with narrow RIPA, and renal anatomy. The total score ranges from zero to four. In our study, the AUC of the RUSS score was 0.828 (95% CI: 0.759-0.897). Erbin et al. assessed the predictive value of the RUSS score in determining SFR and found it to be an independent predictor for SFR (AUC: 0.65 95% CI: 0.589-0.721) [29]. Tufano et al. validated the RUSS score in predicting outcomes after RIRS and found it to have a high diagnostic accuracy (AUC: 0.76) [30].

RIRS scoring system is an innovative scoring system comprising renal stone density, inferior pole stone with RIPA >/<30°, RIL, and stone burden [9]. The score is calculated as four to five (mild), six to eight (moderate), and nine to 10 (severe). It was found that with increasing scores, the SFR decreased. The diagnostic accuracy of this score was reasonably high, with an AUC of 0.882 (95% CI: 0.828-0.936). Our findings are in agreement with a study by Elmohamady et al. in which the RIRS score had a satisfactory AUC of 0.868 [6].

Our study has some limitations. It is recognized that a plain CT scan is the best method to document stone clearance due to its inherent high sensitivity. We did not use CT scans to document SFR for every case. We performed USG to document clearance when a CT scan was not performed. Concerns regarding radiation exposure and the high cost of CT scans led us toward this approach. Ours was a single-center study. Although it adds uniformity to the results, results from a multicenter study may have been different. Another limitation is that we did not measure infundibular breadth and assess the outcome based on it. Furthermore, we did not test stone composition and correlated it with the outcome.

## Conclusions

RIRS has lately emerged as an effective and reliable modality for treating selected renal stones. Patients with large or multiple stones require follow-up due to the high risk of residual stones after a single session of RIRS. Patients who undergo RIRS in large-volume stones should be counseled for staged procedures beforehand. Lower pole stone location, stone density (HU), and abnormal renal anatomy are essential predictors for SFR after RIRS. Lower pole RIPA and RIL are significant influencing factors for SFR after RIRS. RIRS and RUSS scores show a significant association with stone-free outcomes, with higher scores predicting poorer SFR. RIRS score performed better than the RUSS score in predicting stone-free outcomes. These scoring systems can be used preoperatively to gauge treatment success and counsel patients regarding appropriate treatment modalities.
